# Can bupropion unmask psychosis

**DOI:** 10.4103/0019-5545.44907

**Published:** 2009

**Authors:** Sandeep Grover, Partha Pratim Das

**Affiliations:** Department of Psychiatry, Postgraduate Institute of Medical Education and Research, Chandigarh-160 012, India

**Keywords:** Bupropion, bipolar depression, psychosis

## Abstract

Bupropion is an antidepressant, which has recently been promoted for the treatment of bipolar depression, because of its lower potency to induced switch. However, due to its dopamine enhancing effect, it has been reported to induce psychosis and perceptual changes. Most of the literature, which is available in relation to development of psychosis while receiving bupropion, has been with the use of immediate release formulation. Some of the case report which have reported development of psychosis with sustained release Bupropion, it has been reported in the background history of over dosage or substance abuse. We present case in which use of bupropion led to development of frank psychosis, which responded to use of antipsychotic medication. However, when antipsychotics were stopped, psychosis again recurred and as a result diagnosis of patient was changed.

## INTRODUCTION

Bupropion selectively inhibits the neuronal uptake of dopamine and noradrenaline; the dopamine enhancing effect[1] has been implicated for inducing perceptual changes[2] and psychosis.[3,4] The bupropion induced psychosis has been linked to risk factors like old age, past history of psychosis, bipolar disorder, concomitant psychostimulant abuse, rapid increase in dose, large total dose, disturbed liver function tests and concurrent use of other dopaminergic medications.[3–6]

Most of the available literature on bupropion-induced psychosis has been associated with the use of immediate release formulation except for 2 case reports in which development of psychosis was associated with the use of sustained release preparation.[4,6] One of these two cases involved bupropion overdose[6] while the other had a history of alcohol and cannabis abuse.[4] We report a female with bupropion-induced psychosis with therapeutic dose (300 mg daily) of sustained release formulation.

## CASE REPORT

A 36-year-old married homemaker from a Hindu nuclear family who was on lithium 900 mg daily for her DSM-IV diagnosed Bipolar Affective Disorder (NOS) for last 7 years presented to the outpatient department with a 4 week acute-onset episode precipitated by an altercation with the neighbor and characterized by sadness of mood, anhedonia, easy fatigability, poor interaction, disturbed sleep, reduced appetite, low self-esteem, ideas of hopelessness and worthlessness, and marked psychosocial dysfunction.

Her past history revealed that her illness had started after her first childbirth as a postpartum major depressive episode. She improved over a 4-week period with Escitalopram 10 mg daily, which was maintained for a year. When depression relapsed on stopping Escitalopram, it was re-instituted and maintained for the next 3 years. After this point her medication compliance became poor. On one occasion within 3 weeks of reinstitution of Escitalopram she developed a manic switch and her diagnosis was revised to Bipolar Affective Disorder (NOS) and she was started on lithium. Regular lithium and renal function monitoring was done prior to the current episode. Premorbidly she had a well-adjusted personality and there was no history of alcohol or substance use. Her mother had a diagnosis of Bipolar Affective Disorder and was receiving Lithium.

Her mental status examination revealed sadness of mood, preoccupation with the precipitating incident, low self-esteem and ideas of worthlessness. Her serum lithium levels were within normal limit. Initially she was managed with lithium (900 mg daily), clonazepam and supportive psychotherapy. Over the next 3 weeks her depressive symptoms worsened. After discussing the pros and cons of starting antidepressant with her family members, she was started on Bupropion sustained release 150 mg daily; after 7 days the dose was hiked to 300 mg daily. Her biofunctions and depressive symptoms improved but she did not reach her premorbid self. In the week following bupropion dose hike, she started behaving abnormally in the form of aloofness, violent behavior, fearfulness, muttering to self, smiling to self, marked disturbed sleep, expressing persecutory and referential delusions, reporting hearing voices passing derogatory comments, and lacking insight. She had marked dysfunction and stopped doing her household work. During this period on 4 different assessments in the outpatient department she did not manifest any sign and symptom suggestive of organicity; her sensorium was clear and she was orientated to time, place and person. Her husband denied any diurnal variation in her symptoms. Her heamogram, liver and renal function tests, and serum electrolytes were within the normal limits. While Lithium and Bupropion were continued at the same doses, Quetiapine 50-150 mg daily was added. Over the next 3 weeks as her symptoms continued to worsen, hence a diagnosis of bupropion-induced psychosis was made. Bupropion was stopped and Quetiapine was increased to 300 mg daily. While on Quetiapine her psychosis resolved, following which tapering off of Quetiapine was started. However, when the patient was on Quetiapine 50 mg/day, her psychosis reappeared and symptoms continued for 4 weeks, following which Quetiapine was reinstituted and her diagnosis was revised to Paranoid Schizophrenia with Bipolar disorder (currently in remission). With reinstitution of quetiapine, her psychotic symptoms improved over the period of 3 months however, she continues to experience the psychotic symptoms off and on in the absence of any mood symptoms for more than 1 year.

## DISCUSSION

Bupropion-induced psychotic have been attributed to inhibition of the reuptake of dopamine into presynaptic neurons and the resulting increase in extracellular dopamine levels.[1] In the index case there was no past or family history of psychosis, no history of substance use, concomitant use of dopaminergic drugs like amantadine or levodopa. Further, there were no psychotic symptoms prior to starting bupropion, and the psychotic symptoms which developed after starting bupropion were not understandable in the light of mood symptoms, the predominant picture being of psychotic but no clear depressive symptoms. All this suggests that the psychosis was related to the use of bupropion. However, the only risk factor for bupropion induced psychosis in her case was of a bipolar illness.[3–6]

Unlike the case report of Wang *et al*,[6] in which psychosis developed in relation to overdose of sustained release bupropion, in the index case psychosis developed while receiving therapeutic dose of sustained release bupropion. Also, unlike in the case of Neumann *et al*,[4] who developed psychosis with sustained release bupropion and had tested positive for drug screen for cannabis, our case had no evidence of substance use.

The psychosis initially responded to Quetiapine, but the symptoms relapsed after tapering off Quetiapine, suggesting that diagnosis was changed to a psychotic illness with affective illness in remission. The switching from affective spectrum to psychotic illness and vice versa has been reported in the literature in the past also.[7,8] In the index case this switch could be attributed to starting of bupropion. It can be hypothesized that the patient had a vulnerability to develop psychosis and bupropion led to unmasking of the same.

Because of its claimed low manic-switch potential bupropion use is increasing in the cases of bipolar depression. In such situations, our case highlights the potential risk of bupropion-induced/ unmasking of psychosis with sustained release preparations as well. Thus, the clinicians should be vigilant about psychosis during initiation and dose hike of bupropion, and when such a subject develops psychotic symptoms which are not understandable in the light of mood symptoms, the first option should to stop bupropion.
